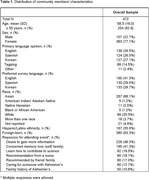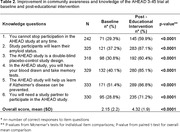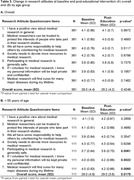# Improvement of Knowledge and Research Attitudes on Preclinical Alzheimer’s Disease Trials through Community‐based Education Among Underrepresented Korean, Filipino, and Hispanic Americans

**DOI:** 10.1002/alz.088665

**Published:** 2025-01-09

**Authors:** Hye‐Won Shin, Melanie B Tallakson, Edwin Duran, Eunji Russ, Dan Hoang, Romina A Romero, David L Sultzer, Joshua D Grill, Christian R Salazar

**Affiliations:** ^1^ Institute for Memory Impairments and Neurological Disorders, University of California, Irvine, Irvine, CA USA; ^2^ School of Medicine, University of California, Irvine, Irvine, CA USA; ^3^ The UC Irvine Institute for Memory Impairments and Neurological Disorders (UCI MIND), Irvine, CA USA

## Abstract

**Background:**

Racial and ethnic minorities, including Hispanic/Latino, Asian American and Pacific Islander individuals, lack adequate representation in preclinical Alzheimer’s disease (AD) trials. This study sought to examine the effectiveness of a culturally and linguistically appropriate community‐based educational intervention in enhancing knowledge and awareness of the AHEAD 3‐45 preclinical AD trial among underrepresented Filipino, Korean, and Hispanic/Latino Americans.

**Method:**

With professional nursing and other partner organizations, we conducted 21 community‐based educational sessions for underrepresented older adults of Filipino, Korean, and Hispanic/Latino backgrounds. In addition to sociodemographic data, we collected the 7‐item Research Attitude Questionnaire (RAQ; Range 7‐35) and a 6‐item Knowledge Questionnaire about the AHEAD 3‐45 preclinical AD trial (Range 0‐6) before and after the educational intervention. Pre‐ and post‐summary scores were generated for both assessments. Paired t‐tests assessed changes in research attitudes and knowledge concerning the AHEAD 3‐45 trial.

**Result:**

Among 654 attendees of the educational interventions, 472 (72%) completed surveys (Table 1). Most were women (77%), immigrants (83%), and had a primary language other than English (70%). The average age was 59 (16) years. Survey responses were obtained in English (41%), Spanish (29%), and Korean (30%). The primary motivations reported by attendees for attending the event were gaining information (48%) and concerns about memory loss for self and/or family members (41%).

Knowledge of the AHEAD 3‐45 preclinical AD trial was significantly increased from a mean of 2.15 (SD 2.2) at baseline to 4.32 (SD 1.9) post‐educational intervention (p < 0.0001, Table 2). Overall RAQ scores remained unchanged for the sample and among Filipino, Korean, and Hispanic/Latino American subgroups (Table 3A), the educational intervention had a positive impact on the research attitudes of attendees under 55 years old (Table 3B)

**Conclusion:**

Our findings indicate the potential effectiveness of culturally and linguistically tailored educational interventions in fostering increased awareness and knowledge of preclinical AD research among Filipino, Korean, and Hispanic/Latino Americans. However, this single intervention did not affect research attitudes overall or in different subgroups most relevant to the AHEAD 3‐45 trial (55 years and older), underscoring the need for additional tailored strategies to enhance representation in preclinical Alzheimer’s disease research.